# Keratin 5 overexpression is associated with serous ovarian cancer recurrence and chemotherapy resistance

**DOI:** 10.18632/oncotarget.14867

**Published:** 2017-01-27

**Authors:** Carmela Ricciardelli, Noor A. Lokman, Carmen E. Pyragius, Miranda P. Ween, Anne M. Macpherson, Andrew Ruszkiewicz, Peter Hoffmann, Martin K. Oehler

**Affiliations:** ^1^ Discipline of Obstetrics and Gynaecology, School of Medicine, Robinson Research Institute, University of Adelaide, Adelaide, 5000, South Australia, Australia; ^2^ Lung Research Laboratory, Hanson Institute, Adelaide, South Australia, Australia, Department of Thoracic Medicine, Royal Adelaide Hospital, Adelaide, 5000, South Australia, Australia; ^3^ Centre of Cancer Biology, University of South Australia and Department of Anatomical Pathology, SA Pathology, Adelaide, 5000, South Australia, Australia; ^4^ Adelaide Proteomics Centre, School of Biological Sciences, University of Adelaide, Adelaide, 5005, South Australia, Australia; ^5^ Department of Gynaecological Oncology, Royal Adelaide Hospital, Adelaide, 5000, South Australia, Australia

**Keywords:** ovarian cancer, keratin 5, tumor progression, recurrence, chemoresistance

## Abstract

This study investigated the clinical significance of keratin 5 and 6 expression in serous ovarian cancer progression and chemotherapy resistance. *KRT5* and *KRT6* (*KRT6A*, *KRT6B* & *KRT6C*) gene expression was assessed in publically available serous ovarian cancer data sets, ovarian cancer cell lines and primary serous ovarian cancer cells. Monoclonal antibodies which detect both K5/6 or only K5 were used to assess protein expression in ovarian cancer cell lines and a cohort of high grade serous ovarian carcinomas at surgery (*n* = 117) and after neoadjuvant chemotherapy (*n* = 21). Survival analyses showed that high *KRT5* mRNA in stage III/IV serous ovarian cancers was significantly associated with reduced progression-free (HR 1.38, *P* < 0.0001) and overall survival (HR 1.28, *P* = 0.013) whilst high *KRT6* mRNA was only associated with reduced progression-free survival (HR 1.2, *P* = 0.031). Both high K5/6 (≥ 10%, HR 1.78 95% CI; 1.03−2.65, *P* = 0.017) and high K5 (≥ 10%, HR 1.90, 95% CI; 1.12−3.19, *P* = 0.017) were associated with an increased risk of disease recurrence. *KRT5* but not *KRT6C* mRNA expression was increased in chemotherapy resistant primary serous ovarian cancer cells compared to chemotherapy sensitive cells. The proportion of serous ovarian carcinomas with high K5/6 or high K5 immunostaining was significantly increased following neoadjuvant chemotherapy. K5 can be used to predict serous ovarian cancer prognosis and identify cancer cells that are resistant to chemotherapy. Developing strategies to target K5 may therefore improve serous ovarian cancer survival.

## INTRODUCTION

Ovarian cancer is the most lethal gynaecological cancer and the sixth most common cause of cancer related death among Western women [1]. Although ovarian cancers represent only 30% of cancers of the female genital tract, they are responsible for half of the deaths [1]. The disproportionately high mortality rate is attributed to the late presentation of the disease. Despite advances in surgery and chemotherapies, no substantial improvement in ovarian cancer survival has been observed over the last two decades [2]. A greater understanding of the mechanisms involved in the progression of ovarian cancer will aid in the discovery of novel molecular prognostic indicators as well as new therapeutic targets. To increase our understanding of the molecular mechanisms involved in ovarian cancer progression and to identify novel therapeutic targets we recently studied the interaction of ovarian cancer and peritoneal cells [3–5]. Keratins K5 and K6c were amongst the proteins that were identified in the ovarian cancer peritoneal cell co-culture secretome by MALDI-TOF/TOF mass spectrometry [5].

Keratins are intermediate filament proteins responsible for structural integrity of epithelial cells and play an important role in epithelial cell protection. They also play roles in cell polarization, cell size regulation, protein translation and organelle positioning [6]. Fifty four functional keratin proteins have been identified in human epithelial cells including 28 type I (acidic forms, K9-K28) and 26 type II (basic forms, K1-K8 and K71-K74) proteins [7, 8]. They contain a central rod of ~310 amino acids with a helical conformation flanked by non-helical head and tail domains of variable length [9]. A characteristic feature of keratin proteins is their pairing with other keratin proteins. They form obligate heterodimers between a type I keratin and a type II keratin via their rod domains and the resulting heterodimers and tetramers form the basic building units of the keratin filaments [7, 8].

K5 (encoded by gene *KRT5*) is a high molecular weight (predicted 62.6 kDa), basic type keratin expressed in the basal, intermediate, and superficial layers of stratified epithelia as well as transitional epithelia and complex epithelium [9]. It is most often complexed with K14 [9]. K5 positive cells have been identified in both luminal and basal epithelium of the normal breast and K5 has been implicated as a stem cell marker [10, 11]. A recent study has highlighted that K5 expressing basal cells in the healthy and regenerating urothelium are self-renewing and unipotent [12].

K6 protein is also a high molecular weight (predicted 60.3 kDa), basic type keratin known to be expressed by proliferating squamous epithelia and usually complexes with K16 [9]. Three isoforms of K6 exist (K6a, K6b, and K6c) which are encoded by three distinct genes: *KRT6A*, *KRT6B*, and *KRT6C* [13, 14]. K6a is the most abundant, representing about 77% of all forms found in epithelia and shares at least 97.6% amino acid identity with other K6 proteins. K6a has been detected in subpopulations of luminal and ductal myoepithelial cells in human mammary glands [15]. A high proliferative population of K6a positive cells has also been described in the prostate gland [16]. There have been only a few studies which have investigated the expression of the K6c isoform in human tissues as until recently there was a lack of isoform-specific gene probes and antibodies.

Monoclonal antibodies to K5 and K5/6 have been used to identify basal-like triple negative breast cancers [17, 18] and high K5/6 expression was found to be associated with an increased risk of breast cancer relapse and death [17, 19, 20]. Focal K5/6 expression has also been described in adenocarcinomas of the endometrium, pancreas and ovary [21, 22]. In addition, K5^+^ subpopulation of cells have been identified in ER^+^ PR^+^ luminal breast cancers [23, 24] and are increased in patients whose luminal breast cancers develop resistance to endocrine treatment and chemotherapy [25, 26].

Whilst other keratins have been shown to have diagnostic or prognostic utility in ovarian cancer [27–30], limited studies to date have examined K5 and K6 expression in this malignancy. We therefore investigated the prognostic significance of *KRT5* and *KRT6* mRNA expression in publically available serous ovarian cancer data sets [31]. Additionally, monoclonal antibodies which detect both K5/6 or only K5 were used to assess protein expression in ovarian cancer cell lines and cohorts of high grade serous ovarian carcinomas at surgery and after neoadjuvant chemotherapy. Furthermore, *KRT5* and *KRT6C* mRNA expression was assessed in chemotherapy sensitive and chemotherapy resistant primary serous ovarian cancer cells derived from patient ascites. We also evaluated whether K5^+^ cells are increased in serous ovarian cancer patients following chemotherapy treatment. To our knowledge, this is the first study to investigate the relationships between *KRT5* mRNA, *KRT6* mRNA, K5/6, and K5 protein expression with serous ovarian cancer patient outcome.

## RESULTS

### *KRT5*, *KRT6C* mRNA and K5/6 protein expression in ovarian cancer cell lines

**Figure 1:**
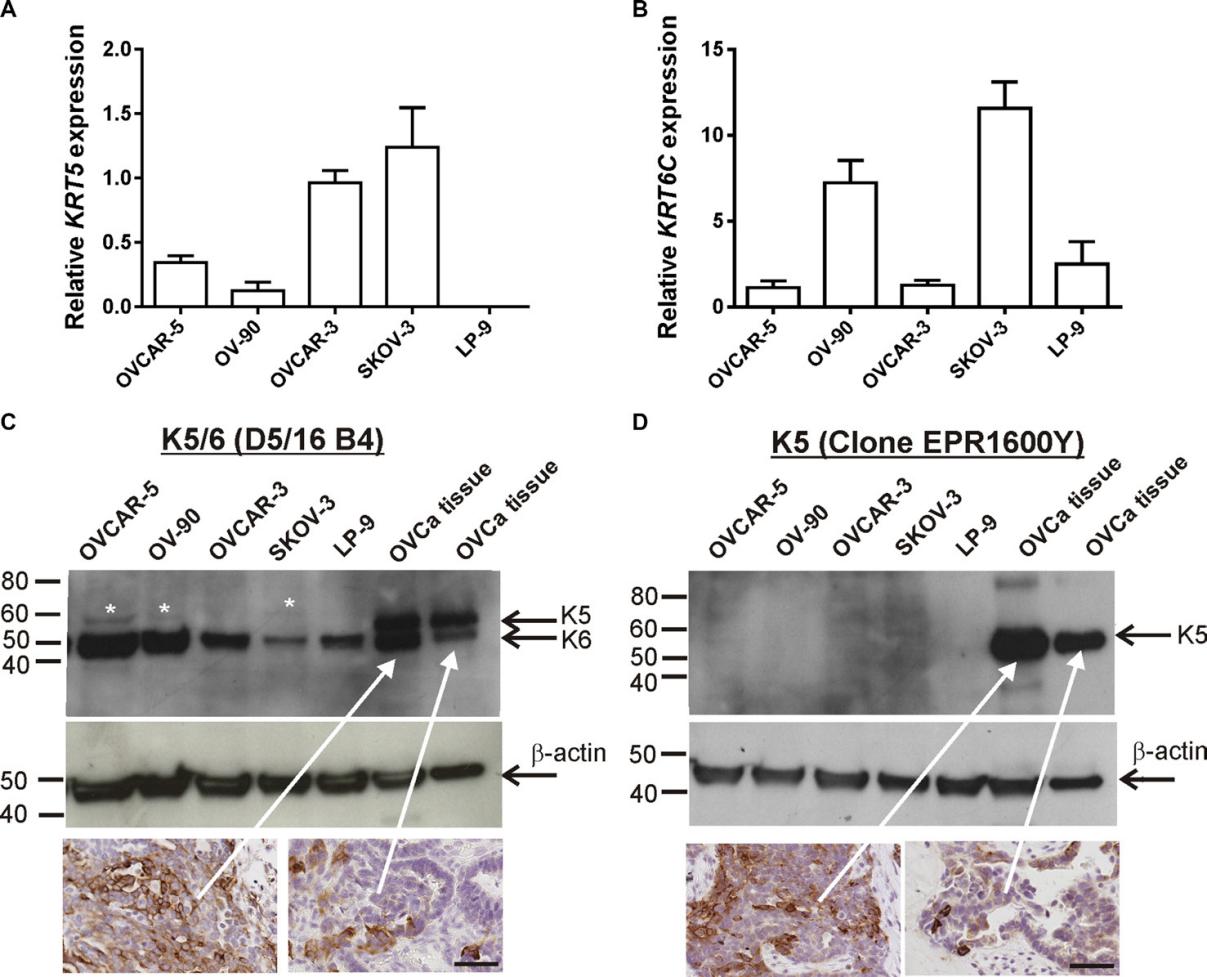
*KRT5, KRT6C* mRNA, K5/6 and K5 expression in ovarian cancer cell lines (**A**) *KRT5* mRNA expression in ovarian cancer cell lines and LP-9 cells. (**B**) *KRT6C* mRNA expression in ovarian cancer cell lines and LP-9 cells. Relative expression was normalized to the house keeping gene β-actin using the 2^-ΔΔCT^ method with the same calibrator (OVCAR-3). Data is expressed as the mean fold change ± SEM from 4-11 individual RNA samples obtained from 2-4 independent experiments. (**C**) Equal amounts protein for the cell lines (40 μg) and ovarian cancer tissue extracts (5 μg) were run on a 4-20% SDS-PAGE gel and immunoblotted with mouse monoclonal K5/6 antibody (1/200, clone D5/16 B4, Dako). K5/6 antibody detected K6 bands at ~52 kDa in all cell line extracts and faint K5 bands at ~56 kDa in cell extracts from OVCAR-5, OV-90 and SKOV-3 cells. Both K5 and K6 were detected in ovarian cancer tissue extracts. White asterisks indicate faint K5 bands detected with K5/6 antibody. (**D**) Western blotting with rabbit monoclonal K5 antibody (1/5000, clone EPR1600Y, Abcam) antibody using the same protein samples from (C) confirmed K5 expression in ovarian cancer tissue extracts. A mouse monoclonal antibody to β-actin (1/10,000, clone AC-15, Sigma Aldrich A3854) was used as a loading control. Ovarian cancer tissues sections with high and low K5/6 (C) or K5 (D) immunostaining were used as positive controls for the western blots (scale bar = 50 μm).

Using qRT-PCR *KRT5* is expressed by metastatic OVCAR-5, OV-90, and SKOV-3 ovarian cancer cells, as well as by poorly metastatic OVCAR-3 cells but not the peritoneal cell line, LP-9 (Figure 1A). *KRT6C* was expressed by all ovarian cancer cell lines as well as LP-9 cells (Figure 1B). The K5/6 antibody detected bands at ~52 kDa in all cell line extracts and faint bands at ~56 kDa in protein extracts from OVCAR-5, OV-90 and SKOV-3 cells (Figure 1C). Using human ovarian cancer tissue extracts shown to express high and low K5/6 and K5 positivity (see inserts in Figure 1C and 1D) and antibodies to only K5, we confirmed that the 56 kDa and 52 kDa bands were K5 and K6, respectively. Two bands at ~52 kDa and ~56 kDa were observed with the K5/6 antibody in the ovarian cancer tissue extracts (Figure 1C), however the K5 antibody (Abcam) only detected a single band at ~56 kDa in the ovarian cancer tissue (Figure 1D).

### K5/6 and K5 are elevated in serous carcinoma tissues

**Figure 2:**
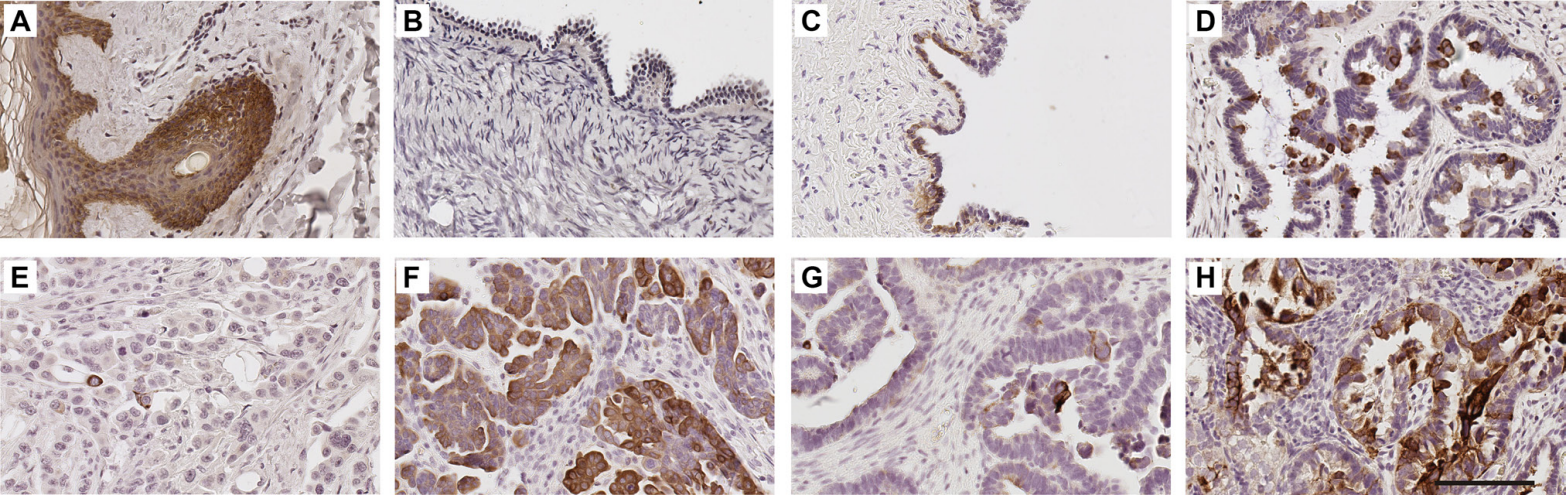
K5/6 expression in human ovarian tissues K5/6 immunohistochemistry using mouse monoclonal K5/6 antibody (1/50, clone D5/16 B4, DAKO) using Tris buffer (pH 9.0) microwave antigen retrieval. Human skin (**A**), normal ovary (**B**), benign serous cystadenoma (**C**), serous borderline tumor (**D**), serous carcinoma with low K5/6 immunostaining (**E**) serous carcinoma with high K5/6 immunostaining (**F**). K5/6 immunostaining in tissues obtained from the same patient at diagnosis (**G**) and following treatment carboplatin and paclitaxel (**H**). Scale bar = 100 μm. All images are same magnification.

K5/6 immunostaining was abundant in the skin epidermis (Figure 2A) but little or no staining was observed in the ovarian surface epithelium (OSE) of normal ovaries (8/8, Figure 2B, Table 1A). High K5/6 immunostaining (score 2 or 3) was present in 25% (2/8) of the benign serous cystadenoma (Figure 2C, Table 1A), 60% (6/10) high grade serous borderline tumors (Figure 2D, Table 1A) and 29.9% (35/117) of the serous ovarian cancer cases (Table 1A). Sixteen percent (19/117) of the serous ovarian cancer tissues were negative for K5/6. Examples of low (score = 1) and high (score = 3) K5/6 immunostaining in serous ovarian cancer tissues are shown in Figure 2E and 2F, respectively. K5/6 immunostaining was increased in serous borderline tumors and serous carcinomas compared to normal ovaries (*P* = 0.006, Chi-Square test, Table 1A). Similar staining patterns were observed with a monoclonal antibody which detects only K5 (Table 1C), (Supplementary Figure 1). However, a higher proportion of serous carcinomas (66%, 70/106) had high K5 immunostaining compared to K5/6 immunostaining. Neither K5/6 nor K5 immunostaining were associated with patient age, FIGO stage, tumor grade or the presence of residual disease (Supplementary Table 1).

**Table 1:** Comparison of K5/6 and K5 immunostaining in different tissues groups

A. K5/6 immunostaining in normal ovaries, benign serous cystadenomas, serous borderline tumors and serous ovarian carcinomas				
Tissue	K5/6 immunostaining (positivity groups)			
	0	1 (1−9%)	2 (10−50%)	3 (> 50%)
Normal ovaries	5/8(62.5%)	3/8(87.5%)	0/8(0%)	0/8(0%)
Serous cystadenomas	0/8(0%)	6/8(75%)	2/8(25%)	0/8(0%)
Serous borderline tumors	0/10(0%)	4/10(40%)	3/10(30%)	3/10(30%)
Serous ovarian carcinomas (Stage III–IV)	19/117(16.2%)	64/117(54.7%)	24/117(20.5%)	10/117(8.5%)
Chi-squared test	*P* = 0.006			

B. K5/6 immunostaining in chemonaive serous ovarian cancers and post chemotherapy treatment			
Tissue	*n*	K5/6 immunostaining (positivity groups)	
		< 10%	≥ 10%
Serous ovarian carcinomas (chemonaïve)	117	82/117(70.1%)	35/117(29.9%)
Serous ovarian carcinoma (Post chemotherapy	21	3/21(14.3%)	18/21(85.7%)
Fisher exact test		*P* < 0.0001	

C. K5 immunostaining in chemonaive serous ovarian cancers and post chemotherapy treatment			
Tissue	*n*	K5 immunostaining (positivity groups)	
		< 10%	≥ 10%
Serous ovarian carcinomas (chemonaïve)	106	36/106(34.0%)	70/106(66.0%)
Serous ovarian carcinoma (Post chemotherapy	21	1/21(4.8%)	20/21(95.2%)
Fisher exact test		*P* = 0.007	

### Relationship of *KRT5* and *KRT6* mRNA and K5/6 protein expression with patient outcome

**Figure 3:**
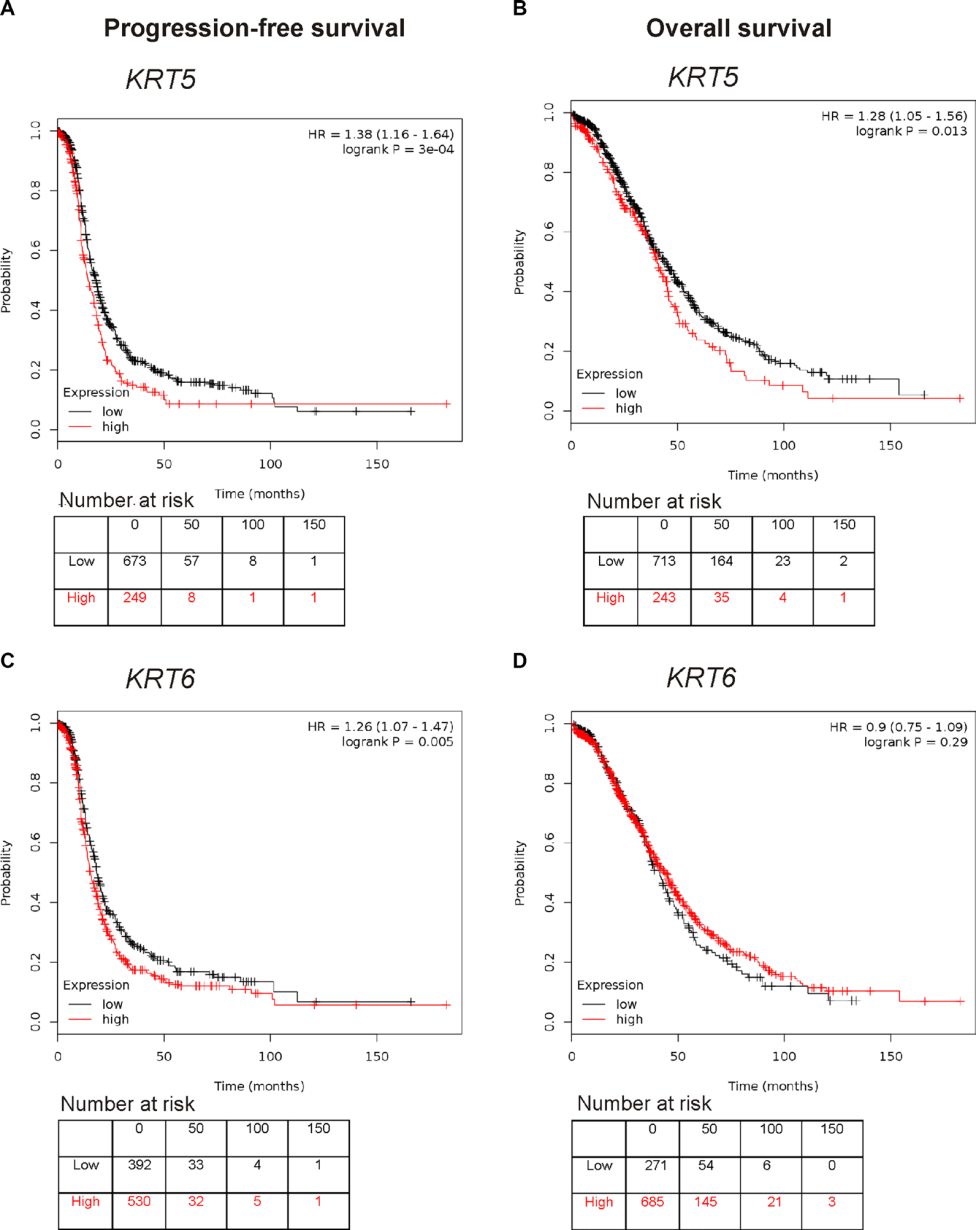
Kaplan Meier survival analysis showing association of *KRT5* and *KRT6* mRNA expression with patient outcome (**A**) Progression-free survival curve in stage III/IV serous ovarian cancers patients with low or high levels of *KRT5* (Affymetrix probe set 201820_at). (**B**) Overall survival curve in stage III/IV serous ovarian cancers patients with low or high levels of *KRT5* (Affymetrix probeset 201820_at). (**C**) Progression-free survival curve in stage III/IV serous ovarian cancers patients with low or high levels of *KRT6* (Affymetrix probeset 209126_x_at, which detects all *KRT6* isoforms). (**D**) Overall survival curve in stage III/IV serous ovarian cancers patients with low or high levels of *KRT6* (Affymetrix probeset 209126_x_at, which detects all *KRT6* isoforms).

Using the publically available Kaplan-Meir online plotter tool which incorporates gene expression data from 13 ovarian cancer sets including the TCGA dataset [31], high *KRT5* expression was associated with reduced progression-free survival (PFS, HR 1.38; 95% CI 1.16–1.64, *P* < 0.0001, Figure 3A) and overall survival (OS, HR 1.28 95%; CI 1.05–1.56, *P* = 0.013, Figure 3B). High *KRT6* expression was also associated with reduced PFS (HR 1.26; 95% CI 1.07–1.47, *P* = 0.005, Figure 3C) but not OS (Figure 3D). No statistical correlation was found between *KRT5* or *KRT6* expression with patient age at diagnosis, tumor stage, tumor grade, or size of residual tumor after cytoreductive surgery in the TCGA dataset (data not shown).

**Figure 4:**
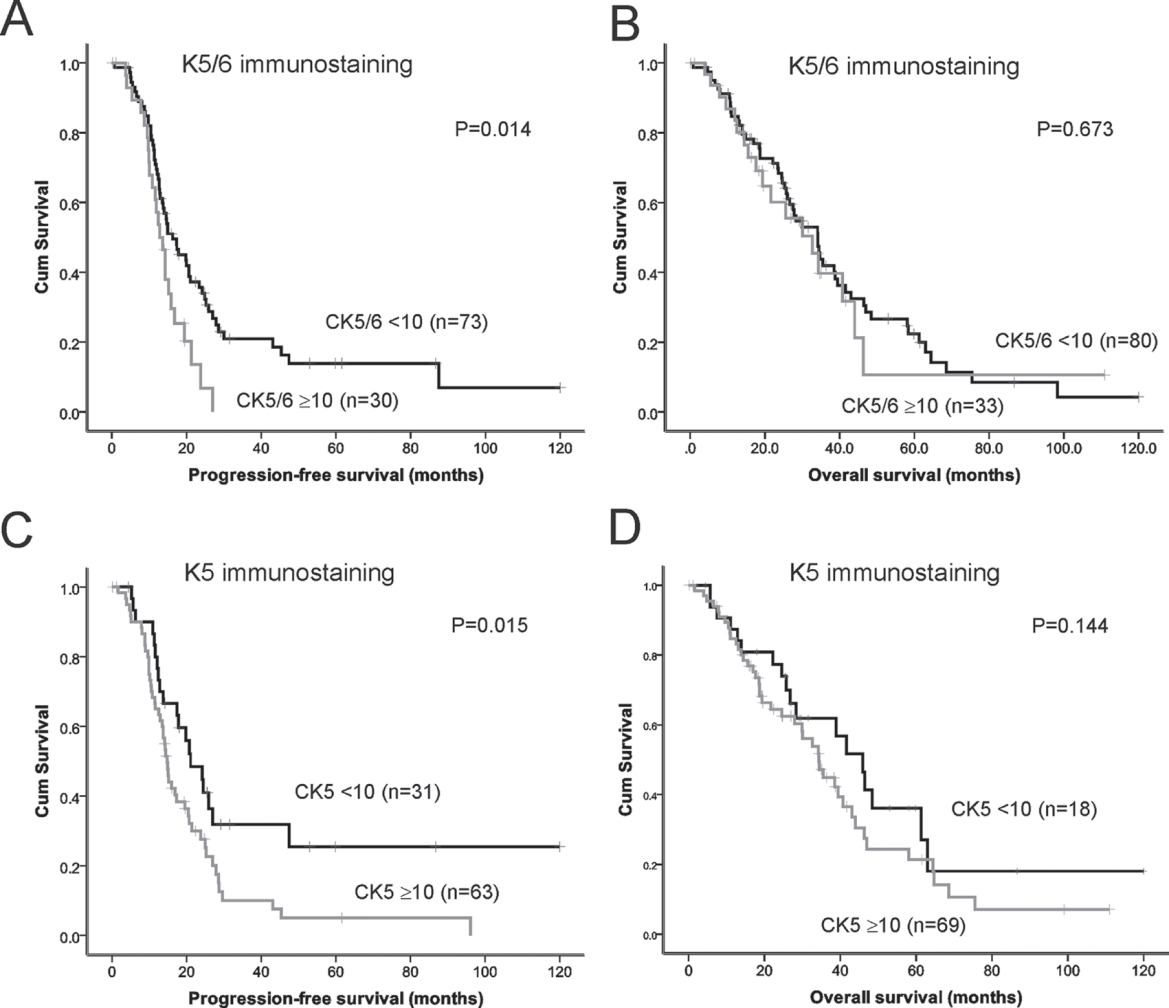
Kaplan Meier survival analysis showing association of expression of K5/6 and K5 alone with patient outcome (**A**) Progression-free survival curve in stage III/IV serous ovarian cancers patients with high K5/6 immunostaining (≥ 10%, *n* = 30) and low K5/6 immunostaining (< 10%, *n* = 73, *P* = 0.014, log rank test). (**B**) Overall survival curve in stage III/IV serous ovarian cancers patients with high K5/6 immunostaining (> 10%, *n* = 33) and low K5/6 immunostaining (< 10%, *n* = 80, *P* = 0.673, log rank test). (**C**) Progression-free survival curve in stage III/IV serous ovarian cancers patients with high K5 immunostaining (≥ 10%, *n* = 63) and low K5 immunostaining (< 10%, *n* = 31, *P* = 0.015, log rank test). (**D**) Overall survival curve in stage III/IV serous ovarian cancers patients with high K5 immunostaining (≥ 10%, *n* = 69) and low K5 immunostaining (< 10%, *n* = 18, *P* = 0.144, log rank test).

We confirmed in a cohort of advanced stage serous ovarian cancers that patients with high K5/6 or high K5 immunostaining (≥ 10.0%) had a significantly reduced PFS compared to patients with low K5/6 or low K5 positivity (< 10.0%, Figure 4A, 4C). The 24 months PFS rate was 34.4% in the group of patients with K5/6 positivity < 10% and only 10.5% in the group of patients with K5/6 positivity ≥ 10%. The 24 months PFS rate was 48.5% in the group of patients with K5 positivity < 10% and only 27.7% in the group of patients with K5 positivity ≥ 10%. Neither K5/6 nor K5 immunostaining was associated with OS (Figure 4B, 4D). Cox regression analysis also indicated that patients with high K5/6 positivity (≥ 10%) had a 1.78 fold increased risk of disease relapse (95% CI; 1.01–2.66, *P* = 0.017, Table 2). High K5 positivity was associated with a 1.90 fold increased risk of disease relapse (95% CI; 1.12–3.19, *P* = 0.017, Table 2). Other clinical and pathological parameters including patient age, clinical stage, tumor grade, and the presence of residual disease were not associated with PFS or OS in this advanced stage serous ovarian cancer cohort (Table 2).

**Table 2:** Univariate cox regression analyses for progression-free survival and overall survival

Progression-free survival				Overall survival			
Variable	Relative risk	95% CI	*P* value	Variable	Relative risk	95% CI	*P* value
Age^a^(*n* = 112)	1.23	0.80–1.90	0.340	Age (*n* = 120)	1.25	0.79−1.97	0.342
Tumor stage^b^(*n* = 112)	0.24	0.19–1.50	0.540	Tumor stage(*n* = 120)	0.52	0.26−1.99	0.718
Tumor grade^c^(*n* = 112)	0.89	0.57–1.62	0.962	Tumor grade (*n* = 122)	0.96	0.57−1.70	0.985
Residual disease^d^(*n* = 89)	1.47	0.75–2.88	0.260	Residual disease(*n* = 96)	2.20	0.99−4.90	0.053
K5/6^e^(*n* = 102)	1.78	1.01–2.66	0.017	K5/6 (*n* = 106)	1.02	0.60−1.74	0.929
K5^f^(*n* = 91)	1.90	1.12–3.19	0.017	K5(*n* = 99)	1.40	0.82−2.44	0.234

NOTE: *P* values highlighted in bold indicate *P* < 0.05.

a Age a dichotomous variable, cut point < 55 vs ≥ 55.

b Tumor stage (FIGO stage III vs FIGO stage IV).

c Tumor grade (moderate vs poor).

d Residual disease status (negative vs positive).

e K5/6 (% positive cells) as a dichotomous variable, cut point < 10 vs ≥ 10.

f K5 (% positive cells) as a dichotomous variable, cut point < 10 vs ≥ 10.

### *KRT5* mRNA and K5 protein levels are elevated following chemotherapy treatment

**Figure 5:**
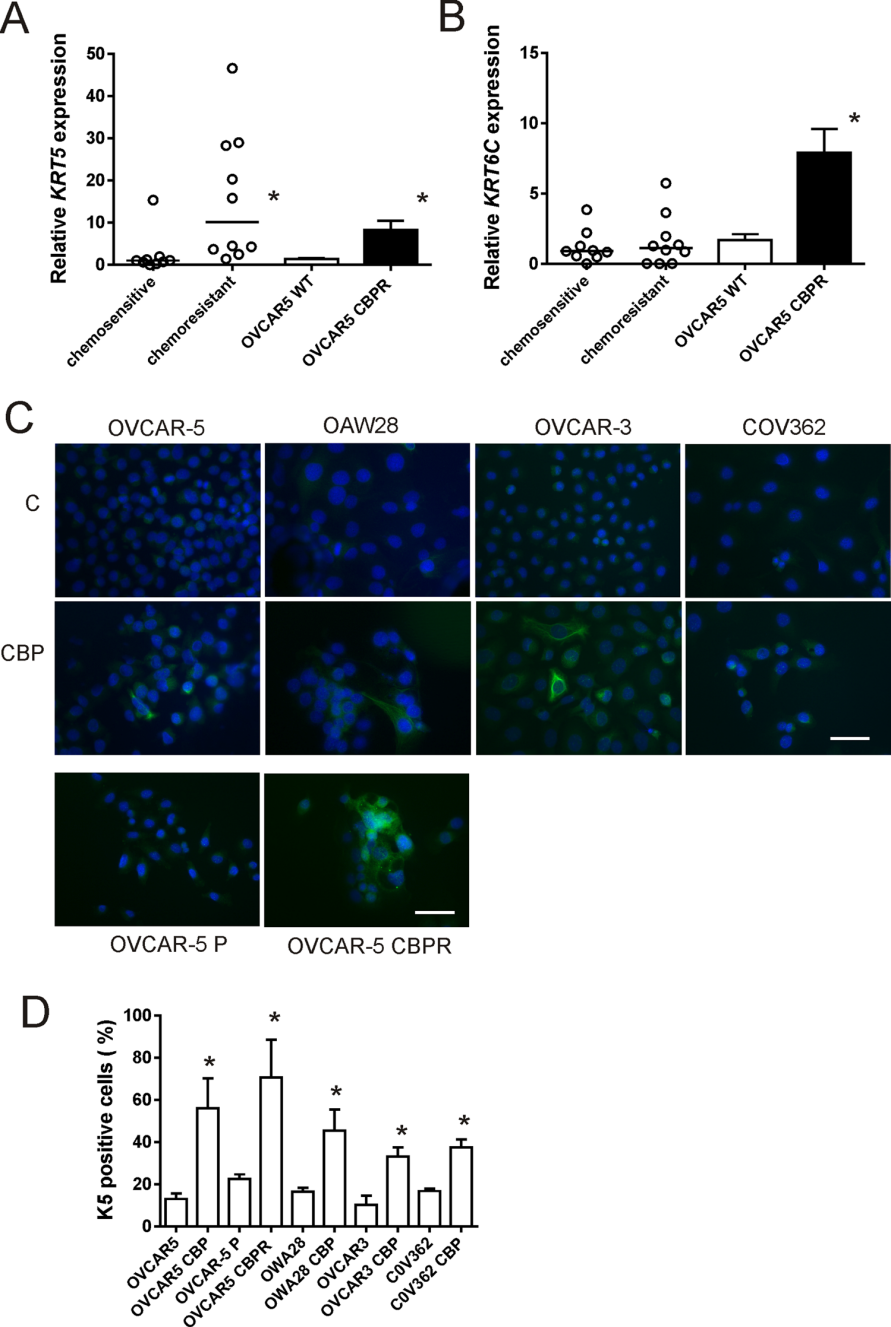
*KRT5, KRT6C* mRNA and K5 protein expression in serous ovarian cancer cell lines following chemotherapy treatment *KRT5* (**A**) and *KRT6C* (**B**) expression in chemotherapy resistant primary serous ovarian cancer cells (*n* = 10) compared to chemotherapy sensitive cells (*n* = 9) and OVCAR5 CBPR made resistant to carboplatin (CBP). *KRT5* but not *KRT6C* was significantly increased in chemotherapy resistant cells (*n* = 10) compared to chemotherapy sensitive cells (*n* = 9, *P = 0.0006, Mann Whitney *U* test). Both *KRT5* (*P < 0.0001, Mann Whitney *U* test) and *KRT6C* (*P = 0.0004, Mann Whitney *U* test) were significantly increased in CBP resistant OVCAR-5 CBPR cells compared to parental OVCAR-5 cells. Data for the primary cells is expressed as the mean fold change from 3-6 RNA samples from 2 independent experiments. Data for OVCAR5 cells is expressed as the mean fold change ± SEM from 10 individual RNA samples from 3 independent experiments. (**C**) K5 immunocytochemistry in serous ovarian cancer cells (OVCAR-5, OAW28, OVCAR-3 & COV362) ± 48 hr treatment with CBP IC_50_ and in parental OVCAR5 and CBP resistant OVCAR5 cells (OVCAR5 CBPR). (**D**) K5^+^ cells are increased following 48 hr treatment with an IC_50_ dose of CBP and the development of chemoresistance. *P < 0.05 (unpaired student *t* test), data is expressed as mean % positive cells ± SEM from 3-4 independent experiments.

K5/6 was increased in serous ovarian cancer tissues following chemotherapy compared with chemonaïve tissues (*P* < 0.0001, Table 1B). Increased K5/6 immunostaining is evident in the images in Figure 2G and 2H that are an example of matching tissues from the same patient before and after chemotherapy treatment, respectively. Similar results were observed with the K5 monoclonal antibody (Table 1C, Supplementary Figure 1G and 1H). These findings are supported by mRNA expression studies in chemosensitive and chemoresistant primary ovarian cancer cells. *KRT5* but not *KRT6C* mRNA expression was increased in primary cells derived from patients’ ascites with recurrent chemoresistant disease (*n* = 10) compared with primary cells from patients who responded to the chemotherapy treatment (*n* = 9; Figure 5A and 5B, P = 0.0006, Mann Whitney U test). *KRT5* (Figure 5A), *KRT6C* mRNA (Figure 5B) and K5 protein (Figure 5C and 5D) were increased in OVCAR-5 cells made resistant to carboplatin (OVCAR-5-CBPR) compared with the parental OVCAR-5 cells by immunofluoresence. Furthermore, treatment with an IC_50_ dose of carboplatin significantly increased the number of K5^+^ cells in 4 serous ovarian cancer cell lines (OVCAR-5, OVCAR-3, OAW28 and COV362) (Figure 5C and 5D).

## DISCUSSION

High grade serous ovarian carcinomas account for nearly 70% of ovarian malignancies. They are characterized by high initial chemosensitivity to platinum based therapies, however 75% of patients relapse after treatment and subsequently become chemotherapy resistant [32]. The development of more effective molecularly targeted therapies to improve survival is urgently required. In this study we show that 1) *KRT5* and *KRT6C* are expressed by ovarian cancer cell lines, 2) *KRT5* expression levels predict reduced PFS and OS for serous ovarian cancer patients, 3) *KRT6* expression levels predict reduced PFS but not OS for serous ovarian cancer patients, 4) Both high K5/6 or high K5 positivity in serous ovarian cancers can predict reduced PFS but not OS, 5) K5/6 and K5 immunostaining is increased in serous ovarian cancers following neoadjuvant chemotherapy, 6) *KRT5* expression levels but not *KRT6C* are increased in serous primary cells derived from patients’ ascites with chemoresistant disease and 7) K5 protein expression is increased in serous ovarian cancer cell lines following carboplatin treatment. Our findings indicate that K5 expression could be used to predict serous ovarian cancer prognosis and may be used to identify cancer cells that are resistant to chemotherapy.

K5 is usually detected using a K5/6 combination monoclonal antibody (clones D5/16B4) as it is closely related to K6. Co-expression of K5 and K6 has been reported in a number of different types of neoplasms including basal cell carcinoma [33], prostate cancer [34, 35], ductal breast carcinoma [36–38], mesothelioma [39, 40], lung carcinomas [41], melanoma, basal cell carcinoma, and salivary gland tumors [22]. K5/6 overexpression is associated with poor prognosis of basal-like breast cancers [17, 18, 42] and was found to be an independent indicator of recurrence-free survival and/or OS in breast cancer [17, 19, 20, 43]. The K5/6 monoclonal antibody has been used for the diagnosis of poorly differentiated squamous carcinomas and undifferentiated nasopharyngeal carcinomas [21, 22]. It can distinguish between small cell lung carcinomas which are K5 negative and malignant mesothelioma which are K5 positive [21, 22]. K5/6 has superior sensitivity and reliability in differentiating between benign and malignant prostate glands when compared with K903 (high molecular weight keratins); and [34] it has been used successfully in a five antibody panel (which also targets TRIM29, CEACAM5, SLC7A5, MUC1) to better classify the subtypes of lung carcinoma [44]. The expression of K5/6 together with p63 has also been used to differentiate between adenosquamous carcinomas and adenocarcinomas in pleural effusion samples [45, 46]. Recently, K5 positive basal cells have also been identified as progenitors of bladder cancers [47].

Several studies have investigated the expression of K5/6 in ovarian cancer but to date K5 or K6 expression has not been linked with ovarian cancer outcome. The incidence of K5/6 positivity (29.9%, Table 1B) in our study was similar to that observed in previous ovarian cancer studies which ranged from 25% to 55.4% [22, 48, 49]. However we observed a higher proportion of serous carcinomas (66%, Table 1C) with high K5 immunostaining which is comparable to a recent study reporting 50% K5 positivity in serous ovarian carcinomas [50]. Our finding is in agreement with the study by Bhargava *et al*, 2008 who found that a monoclonal antibody to only K5 (clone XM26) was more sensitive than the K5/6 monoclonal antibodies (clones D5/16B4) in identifying basal-like breast carcinomas and reported a sensitivity of 97% for K5 but only 59% for K5/6 [18]. The K5 antibody (clone EPR1600Y) used in our study is raised to a synthetic peptide in the head domain of keratin 5 whilst the K5/6 antibody clones were raised against purified keratin proteins. It has been suggested that the lower sensitivity of the K5/6 antibodies by immunohistochemistry may be caused by an interference between each other's antigenic binding sites by steric hindrance [18].

Gene expression studies have previously identified only *KRT5* mRNA and not *KRT6* isoforms in normal breast and basal-like breast cancer in humans [11, 21, 51]. Consequently in normal breast tissues and cancer, the K5/6 antibody is thought to target only K5 [52]. We found LP-9 peritoneal cells to express *KRT6C* but not *KRT5*. We confirmed that K6 protein is expressed in ovarian cancer cell lines and LP-9 cells but only faint K5 bands could be detected with K5/6 in OVCAR-5, OV-90 and SKOV-3 cell extracts. The low expression of K5 protein in ovarian cancer cell lines was confirmed by immunofluorescence as only 10–15% of ovarian cancer cells had detectable K5 protein without carboplatin treatment (see Figure 5C and 5D). The observed molecular weights of K5 (56 kDa) and K6 (52 kDa) were close to the predicted molecular weight of K5 (62 kDa) and K6 (60 kDa), respectively, and consistent with previous studies that have observed K5 at 56 kDa in rat liver cancer [53] and K6 at 50 kDa in bladder cancer [54].

K5/K14 form the main keratins in keratinocytes of stratified squamous epithelia of the epidermis as well as mucosal non-keratinizing stratified squamous epithelia [9]. K5 is strongly expressed in the undifferentiated basal cell layer which contains stem cells and is reduced in the differentiating suprabasal cell layers [7]. Our immunostaining in human skin using the K5/6 and K5 antibodies concur with this finding. Recent studies have reported that K5/K14 modulates cell proliferation and cell differentiation in the stratified epithelia via the P13K/Akt pathway and K5/K14 negatively regulates cell differentiation via the Notch 1 signaling pathway [55]. Consequently K5/K14 is thought to play an important role in the maintenance of cell proliferation in the basal layer of stratified epithelia. It is likely that K5 regulates similar pathways in serous ovarian cancer cells.

Greater than 50% of ER^+^PR^+^ tumors contain ER^-^PR^-^K5^+^ subpopulations [26] and K5^+^ cells are increased in ER^+^ breast tumors following treatment with neoadjuvant endocrine therapy [25]. ER^-^PR^-^K5^+^ luminal breast cancer cell populations, termed ‘luminobasal’ cells exhibiting enhanced progenitor properties can be induced by progestins, glucocorticoids, as well as mineralocorticoids [26, 56–58] and blocked by anti-progestins and prolactin [57]. Interestingly, these K5^+^ breast cancer cells were found to be less sensitive to 5-fluorouracil and docetaxel in *in vitro* culture and exhibited reduced apoptosis [25]. A recent study investigated metastasis formation in ovariectomized mice injected with luminal breast cancer cell lines and assessed the metastatic process following treatment with estradiol or estradiol + progestin [59]. The untreated ovariectomized mice were metastasis-free until they were supplemented with estradiol or estradiol + progestin. Unlike the parental cells that were predominately ER^+^PR^+^K5^-^ the metastases formed following estradiol or estradiol + progestin contained significantly increased proportions of ER^-^PR^-^K5^+^ cells (6–30%). This finding may have important implications for women on hormonal contraception or replacement therapy who may harbor dormant K5^+^ micrometastases. It has also been suggested that basal-like breast cancers in BRCA1 deficient women may potentially arise from K5^+^ luminal progenitors [23]. Compounds that can effectively target these K5^+^ cells have the potential to improve the outcome of luminal breast cancers and basal-like breast cancers. Targeting K5^+^ cells may also be effective in reducing recurrence in patients with serous ovarian carcinoma. Indeed many similarities have been observed between basal-like breast cancers and serous ovarian carcinoma [60].

A recent study by Corr *et al* (2015) demonstrating that K5^+^ ovarian cancer cells were more resistant to cisplatin-induced apoptosis than K5^-^ cells has suggested that K5 is a marker of a chemoresistant subpopulation of ovarian cancer cells [50]. Their observation that the number of K5^+^ cells increased following cisplatin treatment agrees with the carboplatin data presented in our study. We additionally showed that K5 and K5/6 immunostaining is significantly increased following neoadjuvant chemotherapy treatment and that *KRT5* mRNA is increased in chemoresistant compared to chemosensitive serous primary ovarian cancer. These findings support the notion that K5 plays an important role in the development chemotherapy resistance.

In conclusion, this study found for the first time that serous ovarian carcinomas with increased *KRT5* and *KRT6* mRNA expression, as well as increased K5 or K5/6 immunostaining have an increased risk of disease relapse. K5/6 and K5 expression may therefore be used for predicting the prognosis of serous ovarian cancer patients and to aid patient management. In addition our findings that K5 is increased following carboplatin treatment and in chemotherapy resistance cells suggest that K5 could also be used to identify cancer cells that are resistant to chemotherapy. Developing strategies to target K5 may prevent recurrence and chemotherapy resistance in serous ovarian cancer patients.

## MATERIALS AND METHODS

### Cell culture

The human ovarian cancer cell lines OVCAR-3, SKOV-3, and OV-90 were purchased from American Type Culture Collection (ATCC, VA, USA). OVCAR-5 cells were obtained from Dr Thomas Hamilton (Fox Chase Cancer Center, PA, USA) and the peritoneal cells, LP-9 were purchased from Coriell Cell Repositories (NJ, USA). COV362 and OAW28 were purchased from the European Collection of Cell culture (ECCC). OV-90, OVCAR-3, SKOV-3 and OVCAR-5 cell lines were grown in RPMI 1640 media (cat no. R8758, Sigma Adrich, St Louis, USA) whilst COV362 and OAW28 were grown in DMEM media (cat no. 10567-022, Gibco, Life Technologies, Mulgrave, Vic, Australia). All cell lines are cultured with 10% fetal bovine serum (Sigma Aldrich) and maintained at 37^°^C in an environment of 5% CO_2_. OVCAR-5 cells were made resistant to carboplatin (OVCAR-5 CBPR) following treatment with 8 cycles of carboplatin (CBP, 50 μM, Hospira Australia Pty, Ltd). The OVCAR-5 CBPR cells exhibited an IC_50_ (273 μM) to carboplatin that was nearly 3-fold higher than that for the parental OVCAR-5 cells (99 μM) (data not shown)

Primary ovarian cancer cells were derived from ascites collected from serous ovarian cancer patients after informed consent and with approval of the Royal Adelaide Hospital Human Ethics Committee as described previously [61]. All primary cells were grown in Advanced RPMI 1640 medium (cat no 12633-020) supplemented with 4 mM L-glutamine, 10% FBS (Sigma Aldrich, St Louis, MO, USA) and antibiotics (100 U penicillin G, 100 μg/ml streptomycin sulfate and 100 μg/ml amphotericin B, Sigma Aldrich). Methods were carried out in accordance with the approved guidelines. The clinicopathological characteristics of the patients whose ascites was used to isolate the primary cells are shown in Supplementary Table 2.

### Quantitative real-time PCR

Cells were plated at 5,000 cells in 96 well plates and cultured until confluence for 72–96 hr. Total RNA was isolated and reverse transcribed using the TaqMan^®^ Gene expression Cells-to-CT^™^ kit (Applied Biosystems, Mulgrave, Victoria, Australia), as per the manufacturer's instructions. Briefly, lysis solution with DNAse was added to each well and incubated for 5 min at room temperature. Stop solution was then added to each well and mixed. The lysate (10 μl) was added to a 40 μl reverse transcription master mix and reverse transcribed for 1 hr. Resultant cDNA was stored as 50 μl aliquots at −20°C for qRT-PCR analysis. qRT-PCR reactions were performed on triplicate samples using TaqMan^®^ primer sets for *KRT5* (Hs00361185_m1), *KRT6C* (Hs00752476_s1) using the Quantstudio 12K Flex Real Time PCR System (Applied Biosystems). Briefly, PCR reactions were made up to 10 μl and contained TaqMan^®^ Gene Expression Master Mix (2×), primers for the gene of interest, nuclease free water, and the sample cDNA. PCR cycling conditions were as follows: 50°C for 2 min, 95°C for 10 min (with 40 cycles following of 95°C for 15 sec), and 60°C for 1 min. CT values were normalised to the house keeping gene β-actin (Human ACTB 4333762, Applied Biosystems) and calibrator using the 2^−ΔΔCT^ method.

### Western immunoblotting

OVCAR-5, OVCAR-3, OV-90, SKOV-3, and LP-9 cells a were grown to 80% confluence in 75 cm^2^ flasks (Corning, Sigma Aldrich) and cell extracts were collected. Cells were dislodged using a cell scraper and resuspended in 200 μl of RIPA buffer (1% Nonidet P-40, 1% sodium deoxycholate, 0.1% SDS, 0.15 M sodium chloride, 50 mM Tris- HCL and 1 mM EDTA, pH 8.0 with protease inhibitor) and spun at 7000 rpm (Eppendorf 5424 centrifuge) for 10 min and stored at −20°C. Equal amounts of protein were electrophoresed and transferred to PVDF membranes (GE Healthcare, Little Chalfont, England) as described previously [3]. Proteins bands were detected with mouse monoclonal K5/6 antibody (1/200, clone D5/16 B4, Dako, Glostrup, Denmark) or K5 rabbit monoclonal antibody (1/5000, clone EPR1600Y, Abcam Ab75869, Melbourne, Vic, Australia) with anti-mouse or anti-rabbit IgG peroxidase-conjugated secondary antibodies, enhanced chemiluminescence, and autoradiography as described previously [3]. β-actin mouse monoclonal antibody (1/10,000, clone AC-15, Sigma Aldrich A3854) was used as a loading control. Ovarian cancer tissue extracts prepared in RIPA buffer with high K5/6 and K5 immunostaining used as positive controls for the western blots.

### Tissue cohort

Tissue sections were obtained from formalin fixed paraffin embedded blocks from normal ovaries (*n* = 8), benign serous tumors (*n* = 8), serous borderline tumors (*n* = 10), and primary advanced stage (FIGO stage III/IV) serous ovarian cancers (*n* = 126). The cancer tissues were assembled into tissue microarrays (TMAs, 1 mm triplicate cores) from archived tissue (cancer areas identified by pathologist, AR) obtained from serous ovarian cancer patients diagnosed between 1988 and 2012. An additional 21 tissues were obtained from patients with high grade serous cancer after they had received neoadjuvent chemotherapy. Approval was obtained from the Royal Adelaide Hospital Human Ethics Committee and methods were carried out in accordance with the approved guidelines. Detailed pathological and clinical characteristics of the patient samples are summarized in Supplementary Table 3.

### Analysis of public databases

The Kaplan-Meier plotter tool (http://kmplot.com/analysis/) was used to generate survival curves combining *KRT5* (Affymetrix probe 201820_at) and *KRT6* (Affymetrix probe 209126_x_at detects all *KRT6* isoforms) mRNA data from 13 public ovarian cancer datasets [31]. The Kaplan-Meier analysis was performed on the 2015 version database (*n* = 1648) and patients were split by the best cut-off selected by the online plotter tool [31]. PFS and OS data was available for 922 and 956 stage III/IV serous ovarian cancer patients, respectively. cBioPortal (http://www.cbioportal.org/) was used to assess correlations between *KRT5* and *KRT6* expression levels with clinicopathological parameters features in the TCGA 2011 dataset [62, 63].

### Immunohistochemistry

Immunohistochemistry was performed as previously described [64]. Briefly, tissue sections (5 μm) underwent microwave antigen retrieval for 10 minutes at 100°C in a steam microwave (Sixth Sense, Whirlpool, VIC, Australia) in 10 mM Tris buffer, 1 mM EDTA (pH 9.0). Sections were incubated overnight with mouse monoclonal antibody which detects both K5 and K6 (1/50, clone D5/16 B4, Dako) or K5 rabbit monoclonal antibody (1/400, clone EPR1600Y, Abcam Ab75869), in blocking buffer (5% normal goat serum) at 4°C. Visualization of immunoreactivity was achieved using biotinylated anti-mouse or anti-rabbit immunoglobulins streptavidin-peroxidase conjugate, diaminobenzidine substrate as described previously [3]. Human skin was used as a positive control and negative controls included tissues incubated with no primary antibody or with mouse or rabbit immunoglobulins. Slides were digitally scanned using the NanoZoomer Digital Pathology System (Hamamatsu Photonics, SZK, Japan) and images were collected using NDP view imaging software (NDP scan software v2.2, Hamamatsu Photonics). K5/6 or K5 expression was quantified using a visual grading system based on the extent of staining. Percentage of positive tumor cells was graded on a scale from 0–3; 0 = none, 1 = 1–9 %, 2 = 10–50%, 3 = > 50% by two independent assessors used previously for breast cancer [65, 66]. High K5/6 or K5 immunostaining was defined as ≥ 10% positivity and < 10% was defined as low K5/6 or K5 immunostaining.

### Immunocytochemistry

Ovarian cancer cells (2 × 10^4^ cells/well) were plated in 8 well tissue culture chamber slides (Nunclon^™^ Lab-Tek II Chamber slide, RS Glass Slide, Naperville, IL) in 500 μl 10% FBS RPMI for 24 h and treated for 48 h with IC_50_ concentration of CBP or control media. CBP IC_50_ for serous ovarian cancer cell lines were previously determined to be ~100 μM for OVCAR-5, OVCAR-3 and OAW28 cells and 500 μM for COV362 cells. Cells were fixed with cold 100% methanol (5 min) and cold 100% acetone (3 min), washed with PBS and blocked with 5% goat serum and incubated overnight with rabbit monoclonal K5 (1/50, clone EPR1600Y, Abcam Ab75869). K5 was visualized with goat anti-rabbit Alexa Fluor^®^ 488 (1/200, 1hr at RT, catalogue no. A11034, Molecular Probes, Life Technologies) and slides were mounted with Prolong Gold Antifade Mountant with Dapi (catalogue no. P36941, Molecular Probes, Life Technologies). Cells were viewed with an epifluorescence microscope (BX50, Olympus Australia) and imaged using a 40× objective and a Spot RT digital camera (Diagnostic Instruments, Sterling Heights, MI). Negative controls included rabbit immunoglobulin or no primary antibody. The percentage of K5^+^ cells in controls and following carboplatin treatment were evaluated in 5 high power images (~100–200 cells).

### Statistical analyses

All statistical analyses were performed using SPSS for Windows software (Version 21.0, SPSS Inc., Chicago, IL, USA). Chi-squared test was performed to determine the correlation of K5/6 immunostaining in ovarian tumor tissues with clinical and pathological parameters. The Mann Whitney *U* test was used to assess differences between *KRT5* and *KRT6C* expression in the chemotherapy sensitive and chemotherapy resistant primary serous ovarian cancer cells and the parental OVCAR-5 and carboplatin resistant OVCAR-5 CBPR cells. The one way ANOVA with the Dunnet C Post hoc test was used to assess differences between Z scores for *KRT5* and *KRT6* expression and clinical parameters as data was normally distributed. Kaplan-Meier and univariate Cox Regression analyses were performed to assess the association of K5/6 expression in the advanced stage ovarian cancer TMA cohort with PFS and OS. Relapse or death due to ovarian cancer was used as the endpoint to determine whether *KRT5*, or *KRT6C* expression and K5/6 positivity was associated with PFS or OS. Statistical significance was accepted at *P* < 0.05.

## SUPPLEMENTARY MATERIALS FIGURES AND TABLE



## Abbreviations

CBP: carboplatin
CM: conditioned media
HR: hazard ratio
PFS: progression-free survival
OSE: ovarian surface epithelium
OS: overall survival
TMA: tissue microarray.
