# Supplementary material for: Keratin 5 overexpression is associated with serous ovarian cancer recurrence and chemotherapy resistance
Source: Oncotarget. 2017 Jan 27;8(11):17819–32. doi: 10.18632/oncotarget.14867 (PMC5392289; doi:10.18632/oncotarget.14867)
Supplement: Supplementary file 1 [file oncotarget-08-17819-s001.pdf]

Keratin 5 overexpression is associated with serous ovarian cancer recurrence and chemotherapy resistance

Supplementary Materials

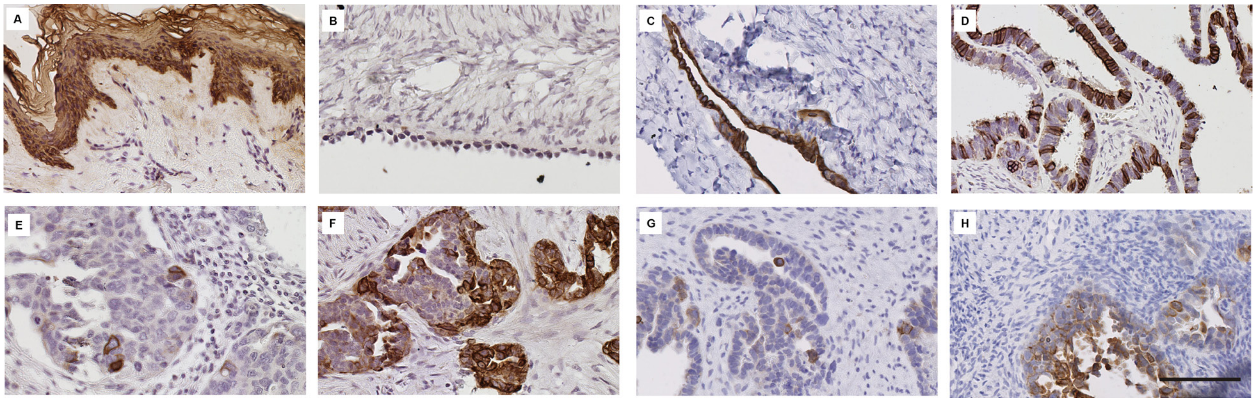

Supplementary Figure 1: K5 expression in human ovarian tissues. K5 immunohistochemistry using rabbit monoclonal K5 antibody (1/400, clone EPR1600Y, Abcam) using Tris buffer (pH 9.0) microwave antigen retrieval. Human skin (**A**), normal ovary (**B**), benign serous cystadenoma (**C**), serous borderline tumor (**D**), serous carcinoma with low K5 immunostaining (**E**) serous carcinoma with high K5 immunostaining (**F**). K5 immunostaining in tissues obtained from the same patient at diagnosis (**G**) and following relapse who was treated with carboplatin and paclitaxel (**H**). Scale bar = 100 µm. All images are same magnification.

Supplementary Table 1: Relationship of K5/6 and K5 immunostaining with clinicopathological parameters

Variable	K5/6 immunostaining		K5 immunostaining	
	< 10% *n* (%)	≥ 10% *n* (%)	< 10% *n* (%)	≥ 10% *n* (%)
Age				
≤ 55	26 (70.3%)	11 (29.7%)	12 (34.3%)	23 (65.7%)
> 55	52 (71.2%)	21 (28.8%)	22 (33.3%)	44 (66.7%)
	$P = 1.00$		$P = 1.00$	
Tumor stage				
FIGO III	77 (71.3%)	31 (28.7%)	31 (32%)	66 (68%)
FIGO IV	5 (71.4%)	2 (28.6%)	3 (50%)	3 (50%)
	$P = 1.00$		$P = 0.394$	
Histological grade				
moderate	11 (55.0%)	9 (45.0%)	2 (14.3%)	14 (85.7%)
poor	71 (74.7%)	24 (25.3%)	32 (36.8%)	55 (63.2%)
	$P = 0.102$		$P = 0.082$	
Residual tumor status				
negative	14 (82.4%)	3 (17.6%)	5 (33.3%)	10 (66.7%)
positive	51 (69.9%)	14 (19.1%)	27 (39.7%)	41 (60.3%)
	$P = 0.379$		$P = 0.773$	

Supplementary Table 2: Summary of clinical and pathological characteristics of the primary ovarian cancer cells established from patient ascites

Patient	Age at Diagnosis (years)	Stage at Diagnosis	Diagnosis	Chemosensitive
1	46	IIIC	Serous papillary carcinoma of the ovary	Yes
2	66	IIIC	Serous carcinoma of ovary/peritoneum	Yes
3	72	IIIC	Serous papillary carcinoma of the ovary	Yes
4	46	IIIC	Serous papillary carcinoma of the ovary	Yes
5	61	IIIA	Serous papillary carcinoma of the ovary	Yes
6	58	IIIC	Serous papillary carcinoma of the ovary	Yes
7	80	IIIC	Serous papillary carcinoma of the ovary	Yes
8	60	IIIC	Serous papillary carcinoma of the ovary	No
9	80	IIIC	Peritoneal carcinoma	No
10	47	IIIC	Serous papillary carcinoma of the ovary	No
*11	59	1A	Recurrent serous tubal	No
12	47	IIIC	Recurrent serous peritoneal cancer	No
13	81	IV	Recurrent serous peritoneal carcinoma	No
14	43	IIC	Recurrent serous peritoneal carcinoma	No
*15	59	IA	Recurrent serous tubal	No
**16	48	IV	Recurrent serous peritoneal	No
17	69	IIIA	Recurrent serous ovarian cancer	No
**18	48	IV	Recurrent serous peritonea	No
19	57	–	Recurrent serous carcinoma	No

*Ovarian cancer cells were derived from the same patient following an interval of 21 months.
**Ovarian cancer cells were derived from the same patient following an interval of 1 month.

Supplementary Table 3: Clinicopathological characteristics of serous ovarian cancer cohort

**Normal ovaries (*n* = 7)**		
Age (years)	Median (range)	45 (39–62)
**Benign serous cystadenomas (*n* = 8)**		
Age at Diagnosis (years)	Median (range)	58 (25–72)
**Serous borderline tumors (*n* = 10)**		
Age at Diagnosis (years)	Median (range)	60 (37–84)
**Chemonaïve serous carcinomas (*n* = 126)**		
Age at Diagnosis (years)	Median (range)	62 (24–87)
Histological Grade	Moderate<br>Poor	21<br>105
FIGO stage	Stage III<br>Stage IV	119<br>7
1st Line Treatment	Cisplatin/cyclophosphamide<br>Carboplatin/paclitaxel<br>Carboplatin alone<br>Other chemotherapy<br>No chemotherapy<br>Unknown	53<br>41<br>7<br>11<br>6<br>8
Residual disease after surgery	No<br>Yes<br>Unknown	18<br>81<br>37
Recurrence	No<br>Yes<br>Lost to follow-up	23<br>92<br>11
Cause of Death	Ovarian cancer<br>Other cause<br>Alive<br>Lost to follow up	83<br>10<br>30<br>3
**Post chemotherapy serous carcinomas (*n* = 21)**		
Age at Diagnosis (years)	Median (range)	68 (50–80)
Histological Grade	Moderate<br>Poor<br>Unknown	–<br>12<br>9
FIGO stage	Stage III<br>Stage IV<br>Not staged	8<br>5<br>8
1st Line Treatment	Carboplatin + Paclitaxel<br>Single Carboplatin<br>Unknown<br>Other	9<br>4<br>7<br>1
